# Role of the Rumen Epithelium and Associated Changes Under High-Concentrate Diets

**DOI:** 10.3390/ijms26062573

**Published:** 2025-03-13

**Authors:** Ling Zhang, Zhenhua Xia, Jicheng Fu, You Yang

**Affiliations:** 1 College of Animal Science and Technology, Southwest University, Chongqing 400715, China; lesley830119@email.swu.edu.cn (L.Z.); zhxia1114@gmail.com (Z.X.); 2 College of Pharmaceutical Sciences, Southwest University, Chongqing 400715, China; fu1958462087@email.swu.edu.cn

**Keywords:** HCD, rumen epithelium, SARA, protein degradation

## Abstract

Increasing the proportion of concentrate in diets can effectively improve ruminant production, and is therefore widely used. However, high-concentrate diets (HCD) enriched with rapidly fermentable carbohydrates can accelerate the production of lactate and short-chain fatty acids (SCFAs). The accumulation of lactate and SCFAs in the rumen leads to a reduction in rumen fluid pH, potentially resulting in subacute rumen acidosis (SARA), which can decrease dry matter intake (DMI), induce local and systemic inflammation, and cause other negative impacts on the host. The substantial prevalence of SARA attributable to long-term HCD causes considerable economic losses, as it can decrease DMI by up to 20%. Understanding its mechanisms and pathogenesis is essential. The rumen epithelium (RE), which is in direct contact with rumen fluid, is an important tissue in the rumen due to its roles in absorption, transport, and barrier functions. The changes that occur in RE under HCD and the subsequent impacts of these changes are worth exploring. In the short term, HCD feeding promotes RE cell proliferation and upregulates the activity of various transporter proteins, enhancing RE absorption and metabolism. However, with prolonged feeding, these functions of RE are negatively affected, accompanied by the development of inflammation. This review elucidates the structure, the functions, and the responses of RE under HCD, providing a detailed analysis of SARA pathogenesis at the cellular and molecular levels.

## 1. Introduction

The rumen provides a suitable environment for microorganisms to break down plant fiber carbohydrates to produce short-chain fatty acids (SCFAs), which are the main source of energy for ruminants [1]. Most of the SCFAs produced in the rumen can be absorbed and metabolized efficiently by the rumen epithelium (RE), which is therefore an important tissue for ensuring energy intake and preventing ruminal acidification from SCFAs accumulation [2]. In pursuit of high production, rapidly fermentable grains are often added to ruminant diets. High-concentrate diets (HCD) generate a considerable volume of SCFAs, which cause acidity in the rumen. Meanwhile, grains with rapidly degradable starch can also increase the production of lactate [3], which has a lower p*K*a than SCFAs (3.9 and 4.8, respectively), thus further lowering the ruminal pH. Therefore, HCD feeding may increase the risk of the host suffering from subacute rumen acidosis (SARA) [4].

SARA is diagnosed when ruminal pH persistently decreases to 5.8 or below for 3 h [5,6,7]. In an acidic environment, the survival of rumen microorganisms, especially Gram-negative bacteria, is compromised, leading to an elevated concentration of microbe-associated molecule patterns (MAMPs) in the ruminal fluid. Although RE as a barrier can prevent MAMPs from entering the organism, the disruption of the RE under HCD facilitates MAMPs and bacterial translocation. This predisposes ruminants to local and systemic inflammation [8]. Furthermore, the absorption and metabolism of the RE are also compromised under these conditions.

The barrier, immune, absorption, and metabolism functions of RE are intricate and vital, and HCD is thought to be the underlying cause of altered RE functions. However, the specific changes and consequences of RE under HCD, as well as the associated underlying mechanisms, remain incompletely elucidated. This review outlines the current research on RE in response to HCD to elucidate the changes in RE and the potential mechanisms involved.

## 2. Structure and Functions of the RE

**Figure 1:**
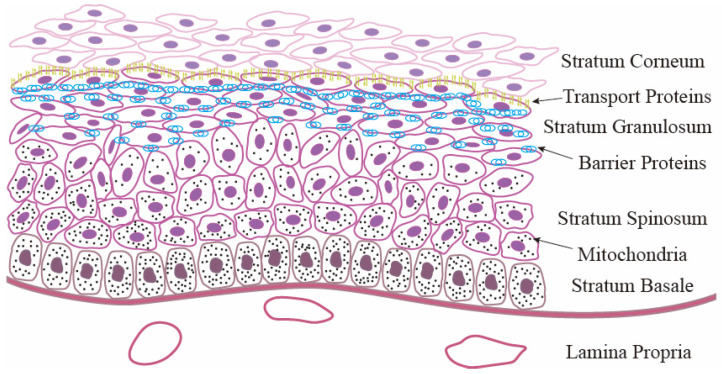
The structure of the rumen epithelium.

Located in the outermost layer of the rumen, the RE is in direct contact with the rumen fluid and plays roles in protection, absorption, transport, and metabolism [9]. RE is composed of the stratum corneum (SC), stratum granulosum (SG), stratum spinosum (SS), and stratum basale (SB) (Figure 1). The surface of the RE is covered with numerous papillae, which help to mix and grind chyme, thereby increasing the interface between chyme and the rumen. The structural attributes of the papillae augment the absorptive surface area, contributing to enhanced absorption and metabolism. Additionally, the papillae serve as sites for the colonization of the microbial community within the rumen.

Tightly attached to the basal lamina, cells in the SB can continuously proliferate, thus becoming a major source of RE renewal and repair. In addition, both the SB and the adjacent SS have functional mitochondria, which help the RE metabolize SCFAs to produce ketones [10]. With cell migration, the number of functional mitochondria in the SG is reduced, and the cell morphology becomes more irregular. Proteins such as claudins, occludin, and zonula occludins are expressed in the granule cells constituting the granule layer, and the density of these proteins decreases towards the SB, indicating the localization of tight junctions (TJ) within the SG [11]. A continuous arrangement of TJ divides the RE into apical and basolateral compartments, providing the basis for the SG barrier function. Furthermore, junctional complexes called desmosomes, located in the SG, help the RE function as a permeability barrier. The uppermost layer of the SG contains various transport proteins that facilitate absorption processes. The SC, with highly keratinized cells, is in direct contact with the rumen fluid and has a large intercellular space, serving as a physical protective barrier for the RE. Therefore, the RE serves as a crucial tissue in maintaining the health and productivity of ruminants. However, the prolonged feeding of HCD exposes the RE not only to essential nutrients, but also to detrimental substances such as bacterial toxins and excessive SCFAs. To ensure the RE retains its beneficial functions, it is imperative that it is not overwhelmed by excessive quantities of highly fermentable carbohydrates, as its physiological capacity is limited, despite its adaptability to dietary challenges [12].

## 3. Responses of the RE Under HCD

### 3.1. RE Proliferation

It has been reported that the short-term feeding of HCD to ruminants does not cause severe damage to the RE [13]. On the contrary, it can promote the proliferation and differentiation of the RE [14]. The reason for this may be that increasing the level of dietary concentrate alters the production and composition of SCFAs, which are gastrointestinal growth factors that effectively stimulate the proliferation of epithelial cells, thus maximizing the nutrient-absorbing surface area of the RE. This finding was confirmed by Bannink et al. [14], whose study showed that epithelial cell proliferation was greater in cows fed on a diet with rapidly increased concentrate percentage after parturition. Appropriately increasing dietary energy levels can elevate rumen SCFAs concentrations. To prevent SCFAs accumulation, SCFAs may regulate the expression of proteins related to the cell cycle, reducing the percentage of cells in the G0/G1 phase and shortening the cell cycle. As a result, this promotes cell proliferation and improves rumen papillae growth [15]. These findings suggest that the RE has a self-regulatory mechanism that can increase or decrease the rate of cell differentiation in response to SCFAs levels. For adapting to an increase in SCFAs, the growth factors bind to receptors on the cell membrane, thereby promoting RE growth and increasing the absorption area. In vitro studies have shown that epidermal growth factor (EGF) produced in the parotid gland may exert a growth-promoting effect by enhancing the transcription of extracellular protein kinases involved in cell proliferation, such as serine/threonine protein kinase (AKT) [16]. EGF is transported to the rumen via swallowed saliva [17], but the expression of EGF in rumen tissue is low due to the lack of physically effective fiber in HCD, which depresses salivation, so EGF may play a small role in the adaptive response of the RE during HCD challenges. Moreover, tissue growth can be promoted by the insulin-like growth factor (IGF) axis-triggered production of protein kinases. Studies have shown that IGF-1 plasma concentration is correlated with rumen papillae growth [18,19]. This may involve two modes of action. One, IGF-1 can upregulate cyclin D1 expression by binding to the IGF-1 receptor, thereby promoting the entry of G0/G1-phase-arrested cells into the S phase [20]. The upregulation of IGF-1 is associated with increased glucose uptake [21], and studies have also shown a correlation between disaccharide level and IGF-1 expression [22]. Thus, increased concentrations of glucose and disaccharides in the rumen derived from HCD degradation appear to be directly related to epithelial cell growth. Two, IGF-binding protein expression is regulated by SCFAs concentration [23]; among them, IGF-binding protein 3 enhances apoptosis by reducing the bioavailability of ligands for the IGF-I receptor [24], while IGF-binding protein 5 acts as a promoter [25]. When ruminants are fed high levels of concentrate, SCFAs downregulate the expression of IGF-binding protein 3 and upregulate the expression of IGF-binding protein 5, thus promoting the proliferation of epithelial cells [26].

RE proliferation under HCD may involve the action of other pathways. For example, HCD have been shown to promote G protein-coupled receptor 41 (GPR41) expression in the rumen, which acts as a receptor for SCFAs [27]; however, by knocking down GPR41 expression in bovine rumen epithelial cells, Meng et al. found that it decreased proliferation by mediating the PIK3-AKT-mTOR signaling pathway [28]. Also, HCD are found to promote RE proliferation through the Hippo pathway [29].

### 3.2. RE Absorption and Metabolism Changes

As previously discussed, the RE can adapt to HCD feeding with an enhanced absorption of SCFAs by promoting the proliferation of the epithelium. Additionally, the activity of transport proteins is also upregulated to further promote SCFAs absorption. At physiological pH, SCFAs in the gastrointestinal tract of ruminants predominately exist in their anionic form, which restricts their passive diffusion across cellular membranes. Consequently, SCFAs are primarily absorbed through facilitated transmembrane transport, and the apical uptake capacity of SCFAs by epithelial cells is a key determinant of ruminal pH and susceptibility to SARA [30,31]. An appropriate increase in the level of dietary concentrate, with elevated SCFAs concentration and appropriately reduced pH (pH = 6.8), significantly increases the mRNA expression of SCFAs transporters, including *monocarboxylate transporter 1/4* (*MCT1/4*), *solute carrier family 26 member 3* (*DRA*), *putative anion transporter 1* (*PAT1*), and *anion exchanger 2* (*AE2*) [32]. According to multi-omics results, the daily infusion of butyrate into the rumen of mature goats promotes *MCT4* mRNA expression and enhances SCFAs uptake on the luminal side of the rumen tube [33]. Nakamura et al. [34] reported that butyrate may increase the uptake efficiency of SCFAs by activating the expression of hypoxia-inducible factor 1α and Sp1 through epigenetic modifications, which in turn stimulate the mRNA expression of *MCT4* and its auxiliary protein CD147. Sehested et al. [35] reported that the administration of an additional daily concentrate to dairy cows resulted in a short-term elevation in rumen SCFAs concentration, which increased butyrate translocation rates, as measured in an Ussing chamber, without leading to a corresponding increase in the surface area of the rumen papillae. These findings suggest that the upregulation of transporter protein expression is more sensitive than epithelial cell proliferation in enhancing the absorption of SCFAs.

The upregulation of SCFAs transporters is also accompanied by the upregulation of genes related to the maintenance of intracellular pH, such as the *Na^+^/H^+^ exchanger* (*NHE*) [36]. Yan et al. [32] reported that increased HCD intake resulted in increased mRNA levels of *NHE1* and *NHE3* in goat rumen. This is because when intracellular HSCFAs dissociate, HCO_3_^−^ is exported from the cell in exchange for SCFAs, which decreases the intracellular pH, leading to the upregulation of relevant H^+^ transporters to balance the intracellular pH [37,38]. In addition, a large amount of CO_2_ is produced during rumen fermentation [39,40], which can be dissolved in rumen fluid, absorbed by the RE, and converted to HCO_3_^−^ through highly active intracellular carbonic anhydrase, increasing the intracellular HCO_3_^−^ concentration and thus buffering the decrease in intracellular pH and protecting the RE [41]. However, whether CO_2_/HCO_3_^−^ can act as a signaling molecule in the regulation of the pathway, leading to protein activation and expression, remains to be investigated.

In addition, an upregulation of *urea transporter B* (*UT-B*) mRNA expression has been observed in the RE of steers fed HCD [42]. When urea is transported into the rumen by UT-B, it can be broken down to produce ammonia, which can combine with H^+^ to generate NH_4_^+^, thereby acting as a buffer against the pH decrease in the rumen. Lu et al. [27] showed that SCFAs and acidic pH cooperatively stimulate mRNA and the protein expression of *UT-B* in rumen epithelial cells (RECs), and demonstrated by in vivo experiments that the concentrations of SCFAs and pH in the rumen are the main factors driving the upregulation of *UT-B* expression. Furthermore, researchers have suggested that the upregulated expression of *UT-B* may be related to the increased expression of *GPR4* and *GPR41*, although the specific mechanisms involved need to be further verified.

However, the increase in absorption does not appear to be sustained for a long period. When energy levels are further increased or the duration of HCD feeding is extended, the combined effects of excessive SCFAs accumulation and the dramatically decreased pH of the rumen eliminate the positive effect of the HCD. Etschmann et al. collected and isolated RE from sheep fed HCD for 0, 1, 2, 4, 6, and 12 weeks, and reported that the HCD increased their net Na^+^ uptake in an Ussing chamber and that 73% of the increase in Na^+^ translocation occurred during the first week of HCD feeding [43]. In ruminants experiencing SARA, an elevated concentration of SCFAs, especially propionate and butyrate, together with a decrease in pH, can downregulate the gene expression of transporters such as *solute carrier family 5 member 8* and *MCT1* [32,44,45], thereby inhibit the uptake of SCFAs, possibly due to the saturation of the metabolic capacity of the RE and the necessity to downregulate transport functions to prevent intracellular SCFAs accumulation.

Despite being the first living cell layer to be exposed to SCFAs, it seems that SCFAs are seldom taken up in the SG, but are mainly taken up in the SS and SB [46]. Although the mechanism remains to be clarified, it seems to be related to the fact that SCFAs are metabolized mainly in the SS. SCFAs metabolism within the RE mainly consists of ketogenic and cholesterol synthesis pathways. Cholesterol is an essential component of mammalian cell membranes; however, an excessive accumulation of cholesterol and its metabolites can trigger inflammatory responses, cell proliferation, and oxidative stress, as well as affect cell membrane permeability [47,48,49,50]. Gao et al. [51] reported that the mRNA level of *hydroxy-3-methylglutaryl-CoA synthase 1*, which catalyzes the metabolism of butyrate in the cytosol to synthesize cholesterol, was significantly greater in cows with a low risk of SARA than in cows with a high risk of SARA. Under short-term HCD feeding, the expressions of genes related to cholesterol synthesis in the RE of lactating dairy cows were increased, and multi-omics analysis showed a strong correlation with rumen fermentation [52]. However, genes related to lipid metabolism and biosynthesis were significantly downregulated in calves under long-term SARA conditions [53]. Steele et al. [54] also reported that genes involved in cholesterol biosynthesis were upregulated in RECs during the first week of HCD feeding but downregulated during the third week. It has been proposed that genes related to SCFAs metabolism are upregulated during the first week due to an increase in the amount of SCFAs substrates used for cholesterol biosynthesis. High intracellular cholesterol concentration is associated with a negative impact; the expression of relevant genes is subsequently downregulated to compensate for the increase in intracellular cholesterol concentration and to maintain intracellular cholesterol homeostasis. Nevertheless, the precise regulatory mechanisms of cholesterol metabolism under HCD feeding need to be further investigated.

To further elucidate the effects of SARA on RE metabolism, the researchers fed one group of cows with a conventional diet and another with HCD, which caused SARA in the latter. Subsequently, they removed the rumen contents of the cows and divided the SARA-affected cows into two groups to facilitate the transplantation of conventional and SARA rumen contents. Transcriptomic and metabolomic analyses revealed that although the recovery of the RE structure was expedited post-transplantation, the functional aspects of absorption and metabolism were not restored [55]. Yang et al. also demonstrated that improved ruminant feeding did not promote the metabolism of SCFAs, although RE proliferation could be accelerated by the activation of Yes1-associated protein 1 and WW domain-containing transcription regulator protein 1 [56]. These studies suggest that the damage to RE metabolism caused by SARA is difficult to reverse.

Interestingly, the transport of SCFAs accumulated under HCD is affected by biological rhythms. Gao et al. [57] reported that RECs under butyrate treatment upregulated the circadian clock gene *period circadian regulator 2* (*PER2*), which caused a decrease in *MCT1/4* expression. Moreover, a reduced expression of *cycles kaput*, which inversely regulates the circadian clock with *PER2* [58], and an elevated expression of *peroxisome proliferator-activated receptors* (*PPARs*) were both shown to be associated with the downregulation of transporter proteins. However, it is unfortunate that the regulatory mechanisms have not been investigated yet.

Finally, RE inflammation caused by HCD-induced SARA can also affect metabolism. Xue et al. [46] indicated that after exposure to lipopolysaccharide (LPS), the consumption of butyrate and propionate is not affected, whereas glucose consumption is increased, and the expression of both the steroidogenic enzyme acetyl-CoA acetyltransferase 1 and the ketogenic enzyme 3-hydroxybutyrate dehydrogenase 1 is downregulated, suggesting that RE metabolism may be shifted towards glycolysis or glucose oxidation.

### 3.3. RE Barrier Disruption

As a part of the animal immune system, the epithelial barrier plays an important role in preventing endotoxin translocation, preventing bacteria from entering the portal circulation and creating osmotic concentration gradients. Although the RE can buffer the increased acid production under HCD via self-regulation, the prolonged consumption of HCD predisposes ruminants to SARA. SARA can compromise the integrity and permeability of the RE [59], which leads to long-term RE defects, such as ruminal parakeratosis [60]. When butyrate accumulates in the rumen, it inhibits epithelial proliferation [61]. Using organoid cultures, Zhang et al. demonstrated that butyrate accumulation promoted RECs hyperkeratosis [62]. Butyrate appears to regulate cellular keratinization via the PI3K-Akt signaling pathway and the Wnt signaling pathway, which regulate the expression of oxidative stress-related enzymes. Zhen et al. [33] showed a correlation between antioxidant protein levels and the expression of barrier-associated proteins by multi-omics analysis. However, further verification of these mechanisms is necessary. Studies have shown that prolonged HCD intake can lead to a reduction in the total depth of the SG, SS, and SB layers of the epithelium, in addition to a decrease in the total depth of the RE [15,63]. HCD intake may increase RE permeability by impairing SG cell adhesion and structure. Studies have demonstrated that HCD intake induces the downregulation of desmosomal cadherin expression in the SG [26], leading to impaired epithelial structure and barrier function, and induces the redistribution of TJ proteins, including Claudin-1, Claudin-4, and Occludin in the RE [64]. Once RE barrier disruption is triggered by SARA, its function is difficult to recover. Hu et al. [63] showed that after switching from HCDs to low-concentrate diets (LCDs) for one month and restoring the RE morphological structure, the barrier function was difficult to recover. However, there is a lack of research on the lag of function recovery to morphology recovery.

RE apoptosis is also involved in barrier function impairment. Under HCD, the abundance of RE proapoptotic response-related proteins (cytochrome C, bcl2 associated x) is greater than that under LCDs [65], whereas the abundance of antiapoptotic factor protein (B-cell lymphoma-2) is lower than that under LCDs [15]. Moreover, even though the surface area of RE papillae increases, the upregulation of apoptosis-related protein expression is found under moderate-concentrate diet (MCD) feeding [15]. Since the upregulation of apoptotic proteins under MCDs is accompanied by cell-cycle shortening, it is hypothesized that apoptosis-producing substances could stimulate cell proliferation to compensate for cell loss to reach equilibrium. However, this equilibrium is disrupted by HCD feeding, resulting in RE injury. This hypothesis was supported by the study of Ma et al. [66]. When Ma et al. added thiamine to HCD feed, ribose metabolites, which are required for RNA and DNA synthesis, were increased by promoting the synthesis and activity of TK enzymes, thereby reducing cell apoptosis and promoting cell proliferation, thus mitigating the damaging effects of the HCD on the RE barrier.

At physiological pH, propionate and butyrate promote epithelial barrier protein expression, whereas TJ protein expression decreases at low pH, increasing epithelial permeability [67]. The rumen contains both organic and inorganic acids, and researchers have investigated the effects of an acidic environment caused by the accumulation of different acids on the RE barrier. The effect of an acidic environment caused by inorganic acids on the RE barrier was demonstrated to be mild in the study by Penner et al. [13]. Subsequent studies have also shown that an impairment of barrier function can only be caused by the co-existence of acidic pH and organic acid accumulation [68]. RE integrity and active electrolytic ion transfer efficiency decrease under SARA conditions. However, the negative effect of acidic pH (pH = 5.0) on epithelial integrity is attenuated when SCFAs are absent, suggesting that increasing the proton concentration alone does not lead to RE barrier disruption. This finding may be attributed to the role of SCFAs as proton carriers; they facilitate the transfer of H^+^ ions to the cell membrane, which leads to intracellular acidification, thus burdening the pH regulatory system, inducing osmotic effects (cellular swelling), and causing inflammation [69].

A characteristic feature of SARA is the occurrence of inflammation, which may manifest locally within the RE or systemically throughout the organism in ruminants. Zhang et al. demonstrated that the activation of the MAPK pathway, especially the p38 and JNK pathways, under HCD feeding directly downregulated *Claudin-1* and *Claudin-4* expression in the RE [70]. In addition, it has been shown that LPS can exacerbate TJ protein degradation by increasing the expression of matrix metalloproteinase-9 [71], leading to increased epithelial permeability [72,73]. Liu et al. suggested that the upregulation of local inflammatory factors, specifically tumor necrosis factor-α (TNF-α) and interferon-γ (IFN-γ), in goats fed HCD could act as an endogenous factor altering TJ protein expression in the RE, thereby causing damage to the RE barrier [59]. Despite the lack of studies directly exploring the effects of inflammatory cytokines on rumen epithelial barrier function, Crawford et al. demonstrated that the inflammatory factors TNF-α and IFN-γ directly induce epithelial barrier dysfunction in the small intestine of cows by altering epithelial cell TJ morphology and the rate of cellular renewal, as measured by organoid culture [74]. As a major trigger of rumen inflammation in SARA, the release of endotoxins (e.g., LPS) can stimulate RECs to produce large amounts of reactive oxygen species (ROS), which cause oxidative damage to mitochondrial proteins, DNA, and lipids, thus plays a role in inducing cell apoptosis [75]. Yang et al. [76] showed that HCD feeding modulates mitochondrial dysfunction via hexokinase 2 and activates the NLRP3 signaling pathway, which mediates the inflammatory response and induces hepatic pyroptosis in dairy cows [77].

In addition, other metabolites in the rumen can also have an impact on RE barrier function. Alterations in rumen microbial composition under HCD feeding lead to changes in rumen metabolites, resulting in elevated levels of the endothelial permeability-increasing factor prostaglandin E1 and the byproduct of polyamine synthesis, 59-methylthioadenosine, the accumulation of which results in toxicity, leading to an increase in rumen epithelial permeability [78,79]. Interestingly, studies have also reported that levels of amino acids differ in different SARA-susceptible herds [80]. The rumen microorganisms can use the accumulated SCFAs as carbon sources and nitrogen compounds (such as ammonia) as nitrogen sources to resynthesize amino acids, resulting in an enrichment in the precursors of biogenic amines (L-histidine, L-arginine, L-lysine, and L-tryptophan) in the rumen [80,81,82]. A high concentration of biogenic amines leads to an increased risk of SARA due to their impairment of the TJ of the RE barrier and their proinflammatory effects [83,84].

### 3.4. RE Inflammation

When SARA occurs, there is an upregulation in the production of MAMPs, such as LPS and histamine (HIS) [85,86,87]. Numerous studies have shown that the concentration of free LPS, a major component of the cell wall of Gram-negative bacteria, dramatically increases in the rumen following the death of these bacteria when ruminants are fed HCD [88]. The elevated levels of ruminal LPS can induce the swelling and rupture of RE papillae, causing localized tissue damage and facilitating the translocation of LPS, which may subsequently lead to systemic symptoms. The caspases expressed in the RE, which act as LPS receptors [89], can be activated by direct binding to LPS, leading to the oligomerization and inflammatory necrosis of RECs [65]. In addition, the recognition of LPS and the triggering of molecular signaling pathways are primarily mediated by Toll-like receptor 4 (TLR-4), ultimately leading to the production of proinflammatory cytokines [90,91]. The expression of *TLR-4* in RE has been demonstrated. Kent-Dennis et al. [92] reported that LPS can elicit an inflammatory response by increasing the expression of *TLR-4* and *TLR-2* in RECs. Many studies have shown that some nutritional regulation strategies can reduce the expression of *TLR-4* in RECs; for example, Jiang et al. [93] reported that the addition of quercetin to LPS-treated RECs exerted an anti-inflammatory effect by reducing *TLR-4* expression, which fully proved the inflammatory pathway mechanism of RECs caused by HCD. Upon the activation of *TLR-4*, the inflammatory process can develop via a MyD88-dependent pathway. The expression of *MyD88* is closely related to the abundance of the *Prevotella* family, especially *Prevotella 1*, suggesting again that this Gram-negative bacterial family plays a major role in the development of the inflammatory response associated with HCD [22]. Furthermore, *TLR-4* activation can also trigger type I and type II interferon production via MyD88-independent pathways [94,95]. And notably, the relative expression of IFN-γ is modulated by dietary factors, with a significant increase in IFN-γ expression observed in response to elevated concentrations of Gram-positive bacteria [96]. Interestingly, although HCD have been associated with an increase in other families of Gram-positive bacteria, there is a decrease in *Bacillaceae* in the rumen fluid, which is positively correlated with the levels of butyrate, isobutyrate, benzoic acid, and isovaleric acid [22], and these organic acids could be used to exert an anti-inflammatory effect [97,98].

HIS, a characteristic aberrant metabolite of HCD in the rumen, has been extensively studied for its role in inflammation. The study conducted in bovine RECs revealed that HIS could promote IκB phosphorylation by upregulating IKK β activity [84], thereby activating the NF-κB pathway and subsequently inducing inflammation in RECs. However, most HIS-related studies have focused on the organismal level, with a notable paucity of studies specifically addressing inflammatory pathways in the RE.

Besides LPS and HIS, the role of ruminal metabolites related to HCD in the development of RE inflammation is worth exploring [99]. Lu et al. reported that the immune response was suppressed when animals were fed 35% concentrate, while it was promoted at the 65% concentrate level, the reason for which may be related to altered amino acid metabolism and lipid metabolism [100]. The analysis of the RNA-seq of sheep RE indicated that amino acids play a role in inhibiting the development of inflammation. When a 1:1 ratio of lysine to methionine was added to the diet, this reduced the inflammatory response by regulating intracellular glutathione and cysteine levels, promoting the expression of anti-inflammatory cytokines, and inhibiting the release of pro-inflammatory cytokines [101]. Experiments in the goat caecum have shown that SCFAs can bind to cell-surface GPRs, such as GPR41 and GPR43, which can activate downstream p38 and ERK1/2 via epigenetic modifications, triggering epithelial inflammation [102]. HCD have been shown to promote GPR41 expression in the rumen [27]. By knocking down GPR41 in RECs, chemokine expression is reduced compared with the wild type under SCFAs treatment. Zhan et al. [103] hypothesized that under SCFAs treatment, GPR41 could mediate protective immunity by increasing chemokine expression to recruit polymorphonuclear leukocytes from lamina propria to the RE. However, acidosis pH was not reached in the study, so it remains to be verified whether further inflammation is triggered under SARA. Kent-Dennis’s research also showed the importance of SCFAs in inflammation development. Under LPS treatment in the absence of SCFAs, the expression of the purinergic receptor P2X 7, which is associated with pro-inflammatory responses, is downregulated within the purinergic signaling pathway. Conversely, the expression of CD73 and the adenosine A2b receptor, both associated with anti-inflammatory responses, is upregulated. However, this modulation is weakened after LPS co-treatment with SCFAs, suggesting that the acidic environment plays a role in the further development of RE inflammation [104]. According to the results of the correlation analysis [22], elevated concentrations of d-glucose-6-phosphate and d-fructose-6-phosphate, which are produced by glucose metabolism under HCD, were positively correlated with the development of inflammation. Although these findings have not yet been validated, the research suggested that glucose-6-phosphate dehydrogenase may be involved in the activation of inflammasomes in response to bacterial infections [105]. Consequently, the impact of glucose metabolites under HCD on the inflammatory response warrants further investigation.

Notably, excessive MAMPs concentrations or repeated exposure to inflammatory stimuli can trigger negative feedback mechanisms that induce tolerance, thereby controlling a high expression of proinflammatory chemokines. While a low concentration of HIS significantly activates the NF-κB pathway, a high concentration of HIS has no significant effect on the activation of this pathway [84]. In Kent-Dennis’s study [92], RECs showed a decreased expression of proinflammatory factors following a subsequent treatment with an identical dose of LPS in comparison to the initial treatment. This finding also seems to explain the failure to observe an effect of LPS on *TLR-4* expression in some studies [71], since it is unknown whether the animals used in the studies (including those used to extract primary RECs) experienced SARA. Consequently, it is plausible that these animals may have developed a tolerance to pro-inflammatory inducers. However, this hypothesis remains to be verified.

## 4. Future Directions

With the continual pursuit of economic benefit and animal welfare, preventing and treating nutritional metabolic diseases caused by HCD has become a common goal. The rumen, an important organ responsible for digestive, metabolic, and barrier functions in ruminants, plays an important role in the pathogenesis of HCD-induced SARA. In the context of the development and widespread application of biotechnology, many studies have focused on investigating the roles of and changes in the RE in the pathogenesis of HCD-induced SARA from a molecular biology perspective. Nevertheless, many problems remain to be explored.

(i) Studies on RE barrier disruption under SARA are mainly focused on the mechanism. It remains unclear whether the recovery of RE barrier function may be delayed compared to the morphological recovery after changing the HCD. Understanding this mechanism can optimize the barrier function of the RE and mitigate the detrimental effects of abnormal metabolites associated with HCD on the host.
(ii) As one of the important intermediate products of HCD in rumen fermentation, lactate has pleiotropic properties in the pathogenesis of SARA. Owing to the conversion of lactate into SCFAs in the rumen, its effects on SARA are easily overlooked. Lactic acidosis, which has been proposed thus far, has focused mainly on the impact of lactate on the microbial community in the rumen under HCD. However, some studies conducted on cattle and monogastric omnivores have shown that lactate can cause inflammation in both the gastrointestinal tract and various parts of the body [106,107]. Other studies have shown that lactate can control the differentiation and function of immune cells under inflammatory conditions and inhibit the inflammatory response [108]. Interestingly, our study demonstrated that RECs cultured with varying concentrations of lactate exhibited damage under the pH condition of acidosis, suggesting that lactate contributes to RECs injury. Quiroga et al. also showed that D-lactate induces the secretion of proinflammatory cytokines from bovine fibroblast-like synoviocytes via the PI3K/Akt/HIF-1 and GSK-3β axes and triggers the release of DNA extracellular traps in bovine polymorphonuclear neutrophils, which can lead to inflammation [109,110]. Current research on the role of lactate in inducing damage to the RE is limited.
(iii) An increasing number of studies are investigating the mechanisms of SARA using cell culture techniques. However, significant structural differences between tissues and monolayer cell cultures may obscure some research results. To circumvent the limitations inherent in conventional cell culture methods, three-dimensional cell cultures and organoid cultures should be promoted and applied. The first organoid model of sheep RECs was successfully established by Xu [111]. The first study using rumen organoids was conducted by Zhang et al. [62]. Rumen organoids with internal lumen are expected to facilitate a more accurate simulation of rumen physiology. However, organoid technology has not been widely used in rumen-related research due to technical difficulties, including issues with the integrity of the simulated in vivo environment, the repeatability of the technique, and the lack of a standardized culture process [112,113,114]. Nevertheless, there is still potential for advances in rumen organoid technology. Exploiting further biomaterials to build three-dimensional structures and organoids may be one of our future endeavors.
(iv) In the investigation of gene expression related to rumen function, most studies have predominantly focused on assessing RNA expression levels. Given that proteins perform functional roles within the organism and that RNA must undergo a series of biological processes to be translated into proteins, changes in RNA expression cannot fully represent changes in protein expression levels. Therefore, despite challenges in finding ruminant-specific antibodies, it is imperative that researchers endeavor to measure protein expression levels to enhance the reliability and validity of their results.

## 5. Conclusions

**Figure 2:**
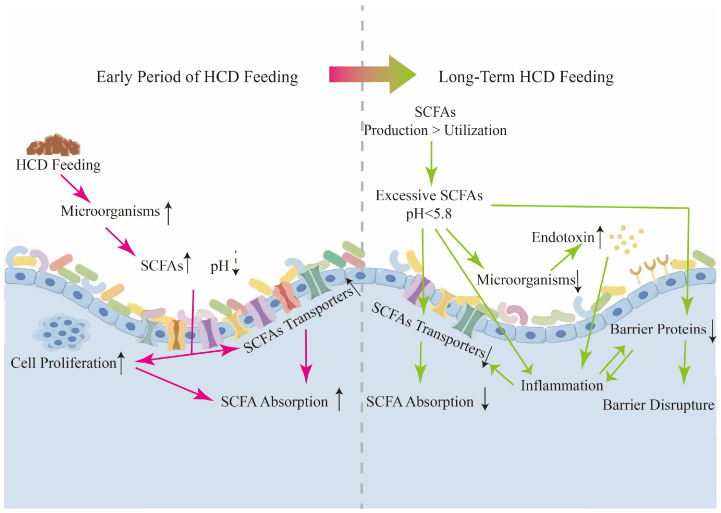
The course of SARA in the RE. HCD: high-concentrate diets; SCFAs: short-chain fatty acids.

To sum up, as illustrated in Figure 2, the RE under HCD feeding can proliferate to expand the absorption area and upregulate the activity of diverse transporter proteins in response to the increase in ruminal organic acids. When the balance between acid production and absorption is disrupted, the RE exhibits an impaired epithelial barrier, suppressed absorption function, and metabolic changes. These changes are all involved in the development of RE inflammation.

## Data Availability

Not applicable.
